# Supplementary material for: Evaluating chatbots in psychiatry: Rasch-based insights into clinical knowledge and reasoning
Source: PLoS One. 2025 Aug 14;20(8):e0330303. doi: 10.1371/journal.pone.0330303 (PMC12352759; doi:10.1371/journal.pone.0330303)
Supplement: Attachment — Submitted filename: Response to Reviewer 2.docx [file pone.0330303.s002.docx]

Reviewer #2: The article is well written.

Thank you for your positive feedback. We appreciate your affirmation.

How was the level of difficulty of the chosen questions from the Taiwan Psychiatry Licensing Examinations assessed to test the range analytic ability of the Chatbots?
Thank you for your question. The Taiwan Psychiatry Licensing Examination has a passing score of 60, with an estimated passing rate of approximately 85% (though this data is not publicly available). While there is no absolute measure of question difficulty, we employed the Rasch model to assess the relative difficulty levels of the selected questions. This method quantifies item difficulty on a logit scale, allowing for a structured evaluation of chatbot performance across varying levels of complexity. Additionally, our approach enables future comparisons with other medical licensing examinations, including those outside of Taiwan, to provide a broader context for chatbot assessment. We have clarified this methodology in the revised manuscript as following: *“Since there is no absolute measure of question difficulty, we employed the Rasch model to assess the relative difficulty levels of the selected questions. This approach also facilitates future comparisons with other medical licensing examinations beyond Taiwan, providing a broader reference for chatbot assessment.”* (Section Statistical Analysis, Page 6, Line 125-128)

What are the limitations of this study?

Thank you for your comment. We have added a dedicated limitations section to explicitly discuss the constraints of this study. We have revised manuscript to provide a more comprehensive discussion, as following: “*This study has several limitations. First, the sample size of 27 chatbots, although sufficient for a pilot study using Rasch analysis [38], may limit the generalizability of our findings. However, the large number of questions used (n = 160) ensured a low standard error for ability estimates, enhancing the reliability of the results. Additionally, our focus on state-of-the-art (SOTA) models may limit the generalizability of our findings to smaller or less advanced language models. Second, while the multiple-choice questions used in this study provided a standardized method of assessing psychiatric knowledge, they may not fully capture the breadth of clinical expertise required in practice. Nevertheless, establishing a clear understanding of chatbots’ knowledge base and reasoning processes remains a fundamental step before integrating them into AI-assisted clinical decision-making, especially given the potential risks associated with their deployment. Third, all questions were presented in traditional Mandarin, and the results may vary across different languages. However, core psychiatric knowledge and reasoning are largely language-independent, as fundamental clinical principles remain consistent across different linguistic contexts. Fourth, while possessing clinical knowledge is important, it does not necessarily translate to the effective application of that knowledge in real-world scenarios. Although we have highlighted key aspects of potential clinical application, future research should prioritize evaluating the efficacy of chatbots in supporting clinical decision-making through controlled trials in real-world psychiatric settings [7, 39].*” (Section Limitations, Page 21, Line 333-352)
