# Supplementary material for: Evaluating chatbots in psychiatry: Rasch-based insights into clinical knowledge and reasoning
Source: PLoS One. 2025 Aug 14;20(8):e0330303. doi: 10.1371/journal.pone.0330303 (PMC12352759; doi:10.1371/journal.pone.0330303)
Supplement: Attachment — Submitted filename: R2_Response to Reviewer 4.docx [file pone.0330303.s003.docx]

Reviewer #4: Well-written article and discussed the main strengths and limitations of current chatbox use in psychiatry. The future directions are well identified and articulated.

Thank you for outlining these thoughtful mitigation strategies. I particularly agree with the importance of prompt engineering—designing structured, context-rich queries significantly improves chatbot performance. This approach helps guide the model’s reasoning process, minimizes ambiguity, and leads to more clinically relevant responses. It's a practical and effective way to align chatbot outputs with expert expectations, especially in sensitive domains like healthcare.

Thank you for this important work.

We are very grateful to reviewer #4 for the positive assessment and encouraging feedback. We are pleased that the reviewer found the article to be well-written and that our discussion of the strengths, limitations, and future directions was well-articulated.

We particularly appreciate the reviewer's validation of our emphasis on mitigation strategies like prompt engineering. This agreement reinforces our conclusion that structured, context-aware interaction is a key method for improving the reliability and clinical relevance of chatbot outputs. It is a critical practical takeaway that we hope our work conveys effectively.
